# 1019. Case Study. Grain Silo Explosion Burn and Blast Injuries: A Five-patient Case Series

**DOI:** 10.1093/jbcr/irag033.344

**Published:** 2026-04-06

**Authors:** Maysa Shemmiyeva, Ariel Santos, Alan Pang, Adel Aziz, Kathleen Seaton, Bernardo Galvan

**Affiliations:** Texas Tech University Health Sciences Center, Lubbock, TX; Texas Tech University Health Sciences Center, Lubbock, TX; Texas Tech University Health Sciences Center, Lubbock, TX; Texas Tech University Health Sciences Center, Lubbock, TX; Texas Tech University Health Sciences Center, Lubbock, TX; Texas Tech University Health Sciences Center, Lubbock, TX

## Abstract

**Patient Presentation (age range, injury details, relevant history):**

Five adult male patients (ages 32–73) were admitted to our regional burn center following a grain silo explosion in March 2025. All sustained mixed partial- and full-thickness burns (10–19% TBSA) to the face and upper extremities, with combined thermal, blast, and inhalation injuries. Bronchoscopy confirmed inhalation injury in all cases. All required early intubation and ventilatory support, with progression to ARDS. Patients had no major comorbidities and were previously healthy manual laborers, emphasizing the sudden, devastating nature of agricultural explosions.

**Clinical Challenges:**

Management was complicated by concurrent burn, blast, and inhalational trauma. Resuscitation required careful fluid titration to avoid worsening blast lung while treating burn shock. All developed ARDS requiring advanced ventilatory strategies. Infectious complications were significant, including ventilator-associated pneumonia, bacteremia, and invasive fungal infections (Aspergillus, Rhizopus). Multiple staged operations (escharotomies, debridements, skin substitutes, STSGs) were performed under physiologic stress. Optimal outcomes required coordination among burn surgery, critical care, infectious disease, respiratory therapy, rehabilitation, and consulting subspecialties.

**Management Approach:**

Treatment emphasized early airway control, bronchoscopy, goal-directed fluid resuscitation, and staged operative management. Aggressive infection surveillance with early broad-spectrum antimicrobial therapy and targeted antifungals was employed. Ventilatory strategies were ARDS-focused, and tracheostomy was performed for prolonged ventilation. Daily multidisciplinary rounds prioritized early mobilization and nutrition to support recovery.

**Outcomes:**

All patients survived to discharge and were transferred to rehabilitation. Median ICU stay was prolonged, with >14 days of mechanical ventilation in all cases and two tracheostomies. Graft take was successful after infection control, and functional outcomes were favorable, with all patients achieving early mobilization and discharge with rehabilitation plans.

**Lessons Learned:**

Early airway control and ARDS management are essential. Aggressive infection surveillance and antifungal therapy are critical in agricultural explosions due to high fungal burden. Staged excision and grafting guided by physiologic readiness improve wound closure. Multidisciplinary coordination and institutional preparedness (ICU surge capacity, OR prioritization, infection control) are key to achieving favorable outcomes, as demonstrated by 100% survival in this series.

**Applicability to Practice:**

This case series highlights key strategies to improve outcomes in burn–blast victims and prepare institutions for industrial mass-casualty events:

-Early Airway & Respiratory Management.

-Robust Infection Surveillance & Control: Routine cultures, strict infection-control protocols, and rapid initiation of targeted antimicrobial and antifungal therapy are critical to reduce sepsis and invasive fungal complications.

-Skin substitutes, and staged STSG optimize graft take and wound closure.

-Multidisciplinary Coordination & Preparedness: Protocols for ICU surge capacity, OR prioritization, and cross-specialty collaboration improve efficiency and patient survival during high-acuity events.

